# DNA Methylation as a Marker of Body Shape in Premenopausal Women

**DOI:** 10.3389/fgene.2021.709342

**Published:** 2021-07-29

**Authors:** Adeline Divoux, Alexey Eroshkin, Edina Erdos, Katalin Sandor, Timothy F. Osborne, Steven R. Smith

**Affiliations:** ^1^Translational Research Institute for Metabolism and Diabetes, AdventHealth, Orlando, FL, United States; ^2^Prescient Metabiomics Corp., Carlsbad, CA, United States; ^3^Department of Medicine, Johns Hopkins University School of Medicine, Johns Hopkins All Children’s Hospital, St. Petersburg, FL, United States

**Keywords:** DNA methylation, fat distribution, adipose tissues, preadipocytes, metabolic risk, blood marker

## Abstract

Preferential accumulation of fat in the gluteo-femoral (GF) depot (pear shape) rather than in the abdominal (A) depot (apple shape), protects against the development of metabolic diseases but the underlying molecular mechanism is still unknown. Recent data, including our work, suggest that differential epigenetic marking is associated with regulation of genes attributed to distinct fat distribution. Here, we aimed to compare the genomic DNA methylation signatures between apple and pear-shaped premenopausal women. To investigate the contribution of upper and lower body fat, we used paired samples of A-FAT and GF-FAT, analyzed on the BeadChip Methylation Array and quantified the differentially methylated sites between the 2 groups of women. We found unique DNA methylation patterns within both fat depots that are significantly different depending on the body fat distribution. Around 60% of the body shape specific DNA methylation sites identified in adipose tissue are maintained *ex vivo* in cultured preadipocytes. As it has been reported before in other cell types, we found only a hand full of genes showing coordinated differential methylation and expression levels. Finally, we determined that more than 50% of the body shape specific DNA methylation sites could also be detected in whole blood derived DNA. These data reveal a strong DNA methylation program associated with adipose tissue distribution with the possibility that a simple blood test could be used as a predictive diagnostic indicator of young women who are at increased risk for progressing to the apple body shape with a higher risk of developing obesity related complications.

**Clinical Trial Registration:**https://clinicaltrials.gov/ct2/show/NCT02728635 and https://clinicaltrials.gov/ct2/show/NCT02226640, identifiers NCT02728635 and NCT02226640.

## Introduction

In 1956 Vague identified central adiposity as a key determinant of several common chronic diseases including diabetes, myocardial infarction, hypertension and stroke. Vague contrasted the disease associated “apple” pattern with metabolically beneficial gluteal – femoral adipose tissue (GF-FAT), or “pear-shape” (Vague, 1956). This is consistent with the concept of lipodystrophy as an etiology of diabetes (Al Ajlouni et al., 2015), where a failure to properly accumulate adipose tissue in the subcutaneous depot is hypothesized to lead to ectopic fat in other locations including the abdominal cavity, liver, muscle, β-cell and brain. In this framework, expansion of GF-FAT prevents visceral and abdominal adipose tissue (A-FAT) expansion by sequestering energy below the waist and away from these deleterious adipose depots and vital organ systems (Manolopoulos et al., 2010). Understanding why GF-FAT can expand in some but not all subjects is essential to explain the overall variation in the susceptibility to develop insulin resistance and/or obesity related comorbidities in women with different body fat distribution. A number of susceptibility genes for preferential fat distribution have been mapped by Genome wide association studies (GWAS) (Dahlman et al., 2016; Lotta et al., 2017). However, they explain only a small portion of the variability and heritability observed in the population. Epigenetic modifications such as DNA methylation and histone modifications have an important influence on gene regulation (Lee et al., 2019) and we hypothesize these difference might explain a significant fraction of the missing heritability.

In this context, we and others showed that A-FAT and GF-FAT have not only distinct phenotypic traits but also differential transcriptomic, DNA methylation and histone mark profiles (Karastergiou et al., 2013; Divoux et al., 2014, 2017, 2018; Passaro et al., 2017). Interestingly, a large fraction of the gene expression differences observed are maintained in isolated preadipocytes cultured from A- and GF-FAT and transferred to the daughter cells, through multiple cell divisions, providing strong evidence for an epigenetic influence to the patterns of differential gene expression (Karastergiou et al., 2013; Pinnick et al., 2014). We also identified distinct chromatin structure signatures in adipocytes and preadipocytes isolated from A- and GF-FAT within the promoter regions of key adipocyte developmental genes (Divoux et al., 2018). More importantly, coupling RNA-seq with the ATAC-seq method we revealed a unique transcriptome associated with specific chromatin openness in A-FAT and GF-FAT between apple and pear-shaped women, suggesting for the first time an epigenetic contribution to the regulation of genes involved in inter individual body fat distribution variation (Divoux et al., 2020).

Here, we tested the hypothesis that DNA methylation profiles of the scWAT depots differs between apple- and pear-shaped women and the differences may contribute to pre-programming each specific fat pattern. Using the Methylation EPIC BeadChip array, quantitatively interrogating over 850,000 methylation sites across the genome, we compared the DNA methylation profiles of 10 apple- and 7 pear-shaped women that were separated based on waist to hip ratio but matched for BMI. It is important to note that all subjects were still relatively young and metabolically healthy. We used paired scWAT samples from the upper and the lower anatomical region (A-FAT and GF-FAT) from each subject. To evaluate the heritability of the body shape specific DNA methylation sites identified in whole tissues, we also compared preadipocytes isolated from the same apple and pear-shaped subjects and cultured through several cell divisions *in vitro*. Finally, we used whole blood samples to evaluate the concordance of DNA methylation between different tissue sources, the overall question being whether DNA methylation profiles in blood can serve as a surrogate for adipose tissues DNA methylation pattern specific to apple vs. pear phenotype. We hypothesized that: (1) there would be differences in DNA methylation profiles in A- and GF-scWAT from apple and pear-shaped women; (2) The differential DNA methylation sites are a part of a stable epigenomic signature that helps differentiate apple vs. pear-shaped women; (3) If the body shape and depot specific differentially methylated sites were found simultaneously in scWAT depots and in blood, the differential signature might serve as a predictive biomarker for future development of the apple vs. pear body shape which would be detectable long before the overt physical differences in appearance and clinical evidence of metabolic disease develop.

## Subjects and Methods

Twenty-one healthy premenopausal, weight-stable females were recruited in Orlando. Participants were further segregated according to their waist to hip ratio (WHR) into two groups: apple-shaped (WHR > 0.85) and pear-shaped (WHR < 0.78). A detailed presentation of the subject characteristics, study design, inclusion and exclusion criteria, body composition, fasting blood results and clinical measurements have been reported previously (Divoux et al., 2020). All procedures were performed under a research protocol approved by the AdventHealth Institutional Review Board.

Adipose tissue biopsies and blood samples were collected as described in Divoux et al. (2018) on a sub-group of 10 apple- and 7-pear shaped women (for 4 women of the initial group, not enough adipose tissue were collected to perform stroma vascular fraction (SVF) isolation and preadipocyte culture as described below). The main clinical data for the 17 subjects are reported in Table 1.

**TABLE 1:** Clinical parameters of 17 women subjects.

**Clinical**	**Pear subjects**	**Apple subjects**	***p*-value**
**parameters**	**(*n* = 7)**	**(*n* = 10)**	
**Adiposity markers**			
BMI (kg/m^2^)	27.4 ± 2.62	28.5 ± 3.74	0.58
Weight (kg)	73 ± 8.8	77 ± 11	0.55
Waist circumference (cm)	80.7 ± 6.03	94.4 ± 8.44	0.003
Hip circumference (cm)	108.1 ± 6.16	107.3 ± 10.5	0.81
Thigh circumference (cm)	60.2 ± 4.58	60.0 ± 5.06	> 0.9
Waist to hip ratio	0.75 ± 0.03	0.88 ± 0.04	< 0.0001
Total Fat mass (kg)	29.2 ± 6.65	33.8 ± 9.54	0.36
Total Lean mass (kg)	41.9 ± 5.4	40.6 ± 3.6	0.67
Fat Mass (%)	39.4 ± 6.5	43.2 ± 6.5	0.27
Android Fat mass (kg)	1.8 ± 0.7	2.9 ± 1.2	0.05
Gynoid Fat mass (kg)	5.8 ± 1.1	6.0 ± 1.8	> 0.9
Leg Fat Mass/Total FM	0.46 ± 0.05	0.36 ± 0.05	0.001
Circulating adiponectin (μg/mL)	13.0 ± 6.0	12.1 ± 5.4	0.87
VAT mass (g)	330 ± 296	724 ± 400	0.04
Liver fat (%)	2.0 ± 1.0	3.0 ± 2.0	0.53
IMAT (cm^3^)	253 ± 113	289 ± 106	0.53

**Lipid profile**			

HDL (mg/dL)	63.1 ± 8.03	63.9 ± 15.2	0.87
LDL (mg/dL)	103 ± 17.5	110 ± 36.6	0.98
VLDL (mg/dL)	14.6 ± 7.72	17.6 ± 5.27	0.11
TGL (mg/dL)	72.3 ± 38.0	88.0 ± 26.2	0.13
Cholesterol (mg/dL)	181 ± 19.3	192 ± 38.2	0.86
Non-HDL chol (mg/dL)	117 ± 22.5	128 ± 38.4	0.87
Chol/HDL	2.93 ± 0.60	3.12 ± 0.78	0.65
LDL/HDL	1.64 ± 0.43	1.84 ± 0.70	0.55
FFA (μmol/L)	458 ± 178	482 ± 145	0.87

**Glucose profile**			

Fasting Glucose (mg/dL)	85.1 ± 6.54	89.1 ± 5.97	0.29
Fasting Insulin [μ(iU)/mL]	6.53 ± 3.71	7.46 ± 3.00	0.40
HOMA-IR	1.38 ± 0.84	1.66 ± 0.72	0.47
HgbA1c (%)	5.33 ± 0.41	5.20 ± 0.24	0.40
C-peptide (ng/mL)	1.20 ± 0.40	1.30 ± 0.38	0.69

**Metabolic markers/hormones**			

Age (years)	33 ± 8.1	35 ± 6.7	0.74
Testosterone (ng/dL)	32.3 ± 21.5	27.8 ± 14.8	0.81
CRP (mg/L)	3.34 ± 3.60	3.02 ± 1.64	0.74

*Mean ± Standard deviation. Non-parametric Mann-Whitney test *p* ≤ 0.05.*

### Adipose Tissue DNA Isolation

Genome DNA was extracted from frozen adipose tissue using the DNeasy Blood and Tissue kit (Qiagen, Valencia, CA), according to the manufacture’s protocol with an additional initial 13,000g centrifugation at 4°C during 10 min to remove the lipids.

### Blood Collection and DNA Isolation

Whole blood was collected after an overnight fast. Blood samples were frozen and stored at −80°C until DNA extraction with the QIAamp DNA mini kit (Qiagen, Valencia, CA).

### Isolation of Preadipocytes and Culture Conditions

SVFs were isolated from scWAT A- and GF-FAT depot, plated and grown in αMEM media supplemented with 2.5% FBS with the addition of hEGF and hFGF (Life Technologies, 10 μg/ml and 4 μg/ml, respectively) during the expansion phase (Divoux et al., 2018). At passage 2, the preadipocyte population was enriched by depletion of the SVF of endothelial cells (CD31 positive cells) using the magnetic beads technology from Stemcell technologies (Cambridge, MA) and samples were frozen for future use. Preadipocytes were thawed, cultured until confluence, harvested, and DNA was extracted with the DNeasy Blood and Tissue extraction kit (Qiagen, Valencia, CA). Another vial of the same preadipocytes were cultured identically and used to perform RNA extraction.

### DNA Methylation

Genome-wide methylation analysis was performed using the Illumina Infinium MethylationEPIC BeadChip platform (Illumina, San Diego, CA), as described in Stephens et al. (2018). Raw data were summarized into BeadStudio IDAT files for further analysis using the Partek Genomic Suite (Partek, Inc., St. Louis, MO). Data were normalized using the SWAN (Subset-Quantile With Array Normalization) method. Differentially methylated CpG sites between apple-shaped and pear-shaped women (or between A and GF depot) were identified by ANOVA using relaxed conditions to define differentially methylated CpG sites. Analyses were performed independently in total adipose tissue, preadipocytes and whole blood samples. Unadjusted *p*-value cutoff = 0.05 was used for body-shape comparison (Apples vs. Pears), adjusted *p*-value cutoff = 0.001 was used for depot comparison (A vs. GF) and unadjusted *p*-value cutoff = 0.05 was used with the blood samples (Apples vs. Pears). ANOVA uses FDR corrected *p*-values. Partek implements the “step up” FDR method by default. For all comparisons, estimated change in β-value cutoff ≥ 0.2 was used. CpG site annotation was based on MethylationEPIC_v-1-0_B4 beadchip from Illumina (species “Homo sapiens,” genome build hg19), using the UCSC database. A maximum distance of 1.5 kb from the methylation locus was considered as a cutoff to determine the related gene. Gene region feature category describing the CpG position were listed from UCSC database using the following parameters: features listed in the same order as the target gene transcripts, TSS200 = 0–200 bases upstream of the transcriptional start site (TSS), TSS1500 = 200–1500 bases upstream of the TSS, 5’UTR = Within the 5’ untranslated region, between the TSS and the ATG start site.

### RNA-seq

RNA was isolated using RNeasy Mini Kit (Qiagen, Valencia, CA) from 17 A-FAT and 17 GF-FAT preadipocytes. RNA-seq was performed as described in Divoux et al. (2018). Briefly, approximately 2 μg was used for library preparation with TruSeq RNA Sample Preparation Kit (Illumina) and libraries were sequenced on an Illumina HiSeq 2500 instrument. The RNA sequencing abundance, raw read counts and TPM values, were calculated with TPMCalculator using the default parameters. The differential gene expression analysis was executed using the Bioconductor packages Deseq2. A *p*-value of 0.05 and a logarithmic fold change of 1.5 was used to identify differentially expressed genes.

### Pyrosequencing Validation

Ten additional healthy premenopausal women were selected from an independent clinical study (Kovacova et al., 2016). Their WHR was not measured during the study. We used the ratio of fat accumulated in the legs divided by the total fat mass obtained by Dexa as criteria to define their body shape. 5 women were considered apple-shaped (44.6 ± 9.4 kg.m^–2^, 39.7 ± 4.8 years, leg fat mass/total fat mass = 0.28 ± 0.03) and 5 women were considered pear-shaped (35.4 ± 3.9 kg.m^–2^, 28.4 ± 7.8 years, leg fat mass/total fat mass = 0.47 ± 0.03). A total of 5 CpG sites, representing 5 individual probes from the Illumina EPIC array were chosen based on their differential methylation status in apples compared to pears. Pyrosequencing method was applied as described in Noble et al. (2021). Briefly, DNA was extracted from total blood samples using the QIAamp DNA mini kit (Qiagen, Valencia, CA), bisulfite conversion was carried out with the Qiagen EpiTect Bisulfite kit, PCR reactions were carried out using the PyroMark PCR kit. Primers to amplify bisulfite-treated DNA were designed using the QIAGEN PyroMark Assay Design Software 2.0. QIAGEN Pyromark Q48 was used to perform the pyrosequencing through the Johns Hopkins Genetic Resources Core Facility (GRCF).

### Pathway Analysis

HypeR, an R package was used for pathway analysis and visualization (Federico and Monti, 2020). Gene set enrichment analysis was performed with hallmark gene sets in Molecular Signature Database (MSigDB). Pathways with *p*-value < 0.05 were marked as significant.

## Results

### Distinct scWAT DNA Methylation Profile Between Apple and Pear-Shaped Women

**FIGURE 1:**
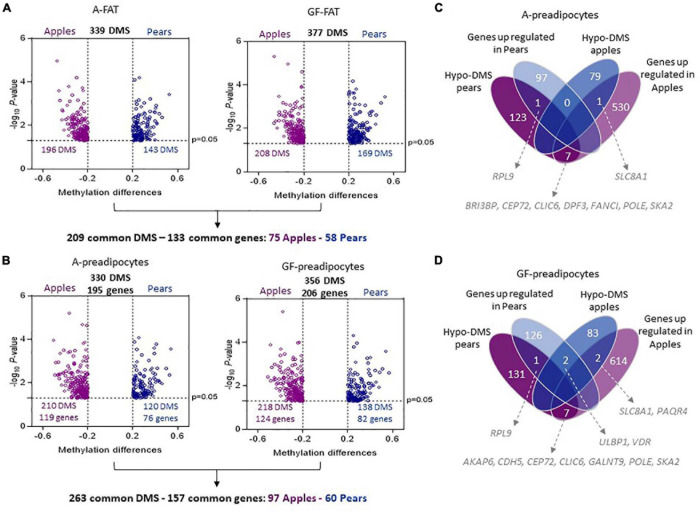
Differentially methylated sites between apple and pear-shaped women in A-FAT and GF-FAT. **(A,B)** Volcano plot showing differences in DNA methylation between 10 apple and 7 pear-shaped subjects in A- FAT (**A**-left side), GF-FAT (**A**-right side), A-preadipocytes (**B**-left side), and GF-preadipocytes (**B**-right side). Each point represents a CpG site significantly differentially methylated with a β-value difference between both groups (methylation difference Apples *vs.* Pears) superior to 0.2. Purple color represents the sites more methylated in Apples. Blue color represents the sites more methylated in Pears. **(C,D)** The Venn diagrams represent the number of differential expressed genes and methylated sites between 10 apples and 7 pears women in A-preadipocytes **(C)** and GF-preadipocytes **(D)**.

We interrogated the level of methylation of 865,918 CpG islands in paired A-FAT and GF-FAT between 10 apple and 7 pear-shaped subjects. To reduce the impact of age and obesity on the level of methylation, both groups of women were matched for age, BMI, weight, total lean mass and total fat mass (Table 1). However, we observed a significant decrease of the android fat mass in pear-shaped women compared to the apple-shaped subjects (Table 1). More importantly, visceral adipose tissue depot size was significantly increased in the apple group (Table 1), suggesting an early default of subcutaneous white adipose tissue (scWAT) lipid storage for this group of women. 339 and 377 sites were differentially methylated between apples and pears in A-FAT and GF-FAT, respectively (Figure 1A, Supplementary Table 1 for the detailed list of DMS). 209 of those differentially methylated sites (DMS), assigned to 133 unique nearest genes based on linear sequence annotation as described in Materials and Methods, were found in both depots, suggesting their contribution in the stable epigenomic structure characteristic of the scWAT (Figure 1A). Importantly, the direction of differential methylation was consistent in both depots for all the DMS. To interrogate these signature genes, we performed gene enrichment analysis and identified intracellular/cytoskeleton and chemokine signaling pathways as specifically enriched in apple-shaped women (normalized enrichment score = 2.16 and 2.04) and interestingly both pathways are relevant to tissue remodeling and inflammation (Trivanović et al., 2018). No significant pathways were enriched in pear-shaped women. Next, we looked at the nearest genes of the body shape specific DMS identified only in A depot (81 genes) or only in GF depot (112 genes). Interestingly, genes involved in glycerophospholipid or lipid metabolism (SCD, TAZ, MBOAT1, LCLAT1, AGPAT2, DGK1, SGMS1) were found only in GF depot, suggesting that the capacity of lipid storage may differ between apple and pear-shaped women in their lower body fat. Extra cellular matrix genes (ABLIM1, PDLIM5) were associated to body shape specific DMS observed only in A depot.

Since adipose tissue is a mixture of different cell types including (but not limited to) adipocytes, preadipocytes, endothelial cells, and immune cells, it is unclear how the distinct cell types contributed to the specific methylome profile we observed between apple and pear-shaped women. Moreover, the proportion of the different cell types varies between phenotype and could influence the methylation differences identified between the 2 groups. Therefore, we decided to explore the CpG island methylation levels in preadipocytes isolated from A-FAT and GF-FAT biopsies from the same 10 apple and 7 pear-shaped women. These cells could differentiate *in vitro* into mature adipocytes and were shown to preserve some of the adipocyte functional characteristics (Lee et al., 2012). The preadipocytes were isolated as described in Materials and Methods and passaged in culture for several rounds of cell division. All the preadipocytes were used at approximately the same passage number (passage 3 or 4). 330 and 356 sites were found differentially methylated between the apple- and pear-shaped subjects in A and GF preadipocytes, respectively (Figure 1B, Supplementary Table 1 for the detailed list of DMS) with a large fraction (43% in A and 41% in GF) located in gene bodies (Supplementary Figure 1). These regions were assigned to 195 unique nearest genes in A-preadipocytes and 206 unique nearest genes in GF-preadipocytes (Figure 1B). 263 common DMS between A and GF cells were nearest 157 unique genes (97 apple specific and 60 pear specific). We sifted these gene sets through the HypeR analysis pipeline to determine which biological processes might be associated with DNA methylation differences. The genes near to the body shape specific DMS in A depot are regrouped under “Allograft rejection” pathway, some inflammatory genes being listed (IL2RA, HLA-A, TLR6, NCF4). Interestingly, the genes near to the body shape specific DMS in GF depot are uniquely regrouped under pathways known to influence adipogenesis (p53, Notch signaling and Pi3k Akt MTOR signaling) (Supplementary Figure 2A).

To get insight into the possible influence of the identified DMS on gene transcription, we performed RNA-seq and pathways analysis on the same preadipocytes isolated from the 17 apple and pear women (at different passage). Out of the 23,511 annotated transcripts, 636 and 752 genes were differentially expressed in apple- compared to pear-shaped cells in A-FAT and GF-FAT, respectively. The lists of differentially expressed genes (DEG) between groups in each fat depot and the list of associated pathways are reported in Supplementary data (Supplementary Table 2 and Supplementary Figure 2B). Interestingly, the most significantly over-represented pathways found in both fat depots are “E2F targets,” “G2M checkpoint” and “mitotic spindle,” these pathways referring to genes that encodes protein involved in DNA replication. Mitotic clonal expansion, including appropriate DNA replication, is required for differentiation of 3T3-L1 (Tang et al., 2003). Moreover, defects in the G2M transition, which is an important cell cycle checkpoint, can lead to cell death (Russo et al., 2016).

The integration of RNA-seq and DMS assigned to the nearest gene showed a relative low correlation between differential gene expression and differential DNA methylation (Figure 1C in A-preadipocytes and (Figure 1D) in GF-preadipocytes), as it has been previously reported in other cell types (Klug et al., 2010; Lim et al., 2017). Only 9 genes in A-cells (Figure 1C) and 12 genes in GF-cells (Figure 1D) are simultaneously differentially expressed between apple and pear-shaped women and have identified DMS within 1,500 bp of their coding sequence. Among these genes, SKA2, upregulated and hypermethylated at its 3′UTR location in apple women (both fat depots), is hypothesized to reduce the ability to suppress cortisol (Kaminsky et al., 2015) and could play an important role in development of visceral adipose tissue which is more prevalent in the apple-shaped women. Enrichment of DNA methylation sites in 3′UTR region have been shown to be uniquely correlated with increased gene expression in T cells (McGuire et al., 2019), suggesting a role of DNA methylation in gene expression activation.

### Conserved DMS in Cultured Preadipocytes and Whole Blood DNA

**FIGURE 2:**
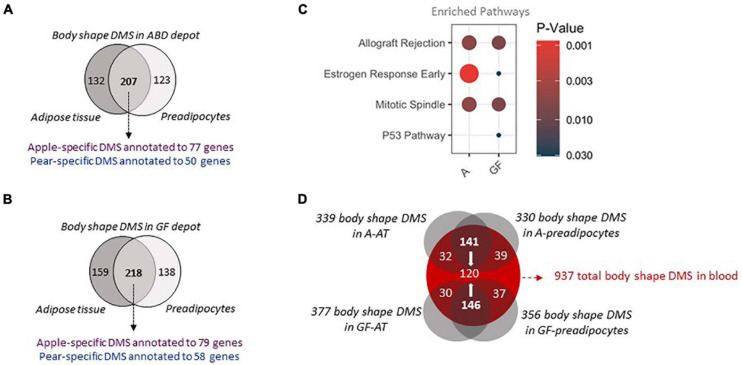
Shared body shape differentially methylated sites between adipose tissue, cultured preadipocytes and whole blood **(A,B)**. The 2 Venn diagrams depict the differentially methylated sites between 10 apple and 7 pear-shaped subjects in A **(A)** and GF **(B)** depot only in adipose tissue, only in preadipocytes or in both. Purple color depicts the number of unique genes annotated to sites hypermethylated in apple-shaped women. Blue color depicts the number of unique genes annotated to sites hypermethylated in pear-shaped women. **(C)** Pathway Analysis of the genes nearest the body shape specific DMS in A depot (A) and GF depot (GF). Only the common genes between whole tissue and cells were used. The size of the dots represents gene count. Only pathways with *p*-value < 0.05 are represented. **(D)** The Venn diagram depicts the common body shape DMS between adipose tissue, preadipocytes and blood for the 17 subjects. A = Abdominal, GF = Gluteo-femoral, DMS = Differentially Methylated Site.

By comparing the DMS from adipose tissue samples with those found in the preadipocytes, we discovered 207 (62%) and 218 (59%) identical DMS between tissue and cells in A-FAT and GF-FAT, respectively (Figures 2A,B). The shared DMS between tissue and cells represent the marks retained after cell isolation and passage in culture which defines them as part of a heritable DMS program conserved from preadipocytes to adipocytes. Pathway analysis revealed that the genes nearest to the body shape specific DMS are enriched for “allograft rejection” “estrogen response” and “mitotic spindle” (Figure 2C), 3 pathways previously identified in our preadipocytes RNAseq and DMS data (Supplementary Figures 2A,B). We then searched for genes that behave in the opposite direction in the 2 fat depots and in the 2 groups of body shape. Interestingly, SPSB4 was the only gene with hypermethylated sites simultaneously in GF-depot of apple women and in A-depot of pear women, both depots that have limited capacity for lipid storage. SPSB4 gene is coding for a protein involved in the regulation of proteasomal degradation and the innate immune system, however its precise function is still unclear (Kleiber and Singh, 2009) and merits further investigation particularly in the context of adipose tissue expansion.

**FIGURE 3:**
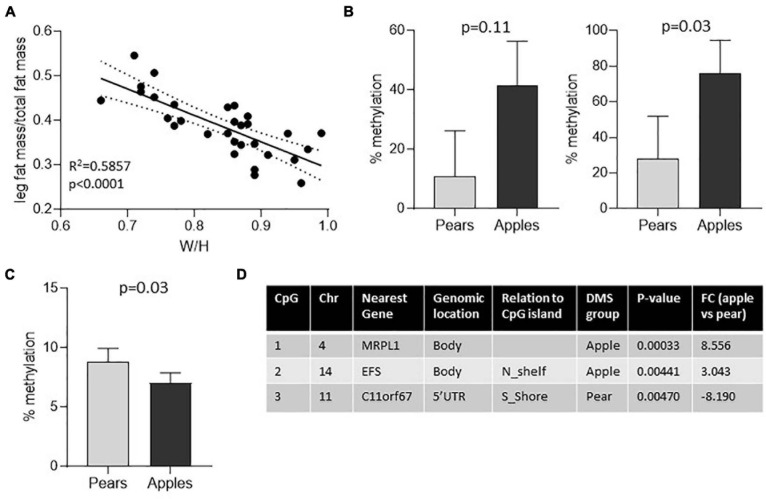
Three of 5 body shape specific DMS found in blood were validated in an independent group of women. **(A)** Linear regression shows positive association between waist to hip ratio and the ratio of fat accumulated in the legs relative to the total fat mass in the initial group of women. **(B,C)** Percentage of DNA methylation in 5 apple- vs. 5 pear-shaped women for 2 apple shaped-specific DMS **(B)** and 1 pear shaped-specific DMS **(C)**. Mean ± SEM is shown. The *p*-value of non-parametric Mann Whitney test are reported Level of CpG sites methylation was measured by pyrosequencing in DNA isolated from total blood samples. **(D)** Genomic characteristics of the CpG sites showing differential level of methylation between apple- and pear-shaped women. Chr, Chromosome, DMS, Differentially Methylated Sites, FC, Fold Change.

Finally, to evaluate whether the conserved DMS between the whole fat and the preadipocytes are unique to scWAT or whether they also may be present in other tissues, we investigated the DNA methylation patterns in matching whole blood samples and compared the DMS found in blood with the DMS identified in adipose samples. Around one third of the body shape DMS identified in the 4 groups of samples (A-FAT, GF-FAT, A-preadipocytes and GF-preadipocytes) were also significantly differentially methylated in DNA isolated from blood (Figure 2D – 120 out of 339, 330, 377, or 356 DMS in fat samples). Overall, these results suggest that the DNA methylation differences associated with apple vs. pear women are not unique to the scWAT depots and there is a more fundamental physiologic difference between the two groups of women and suggests that fat depot non-autonomous events may contribute to at least some of the variation in metabolic disease risk between apple- and pear-shaped women. This also identifies an easily accessible diagnostic signature that may predict cardio metabolic risk early in life before the development of macroscopically apparent body shape differences. To validate the differential methylation signatures, we investigated the level of methylation of 5 body shape specific DMS identified in the first group of subjects in an independent group of premenopausal women. The waist to hip ratio commonly used to determine body shaped were not measured for these subjects. However, the WHR is highly correlated with the percentage of fat accumulation in the legs relative to the total fat mass, both measured by Dexa (Figure 3A). According to this criteria, we selected 5 apple shaped women and 5 pear shaped women from this independent cohort, isolated DNA from whole blood sample and evaluated the level of methylation of the selected DMS by the independent pyrosequencing method. Out of the 5 DMS pre-selected, 2 apple-shaped DMS (Figure 3B) and 1 pear-shaped DMS (Figure 3C) were also found differentially methylated between the apple- (low% of fat in the legs) and pear- (high% of fat in the legs) shaped women. Details about their genomic location and statistics in the initial group of Apple vs. Pear-shaped women are summarized in Figure 3D.

### Preadipocytes Isolated From A and GF-FAT Are Characterized by Differential DNA Methylation Profile, Notably in Apple-Shaped Women

To further examine the epigenetic contribution to apple vs. pear phenotype, we compared the methylome between the 2 scWAT depots (A and GF-FAT) and their isolated preadipocytes. The 2 scWAT shared many properties but there are functional, transcriptional and epigenetic differences between A and GF-FAT that could explain the fat distribution variability observed in premenopausal women. To highlight the unique differences related to a specific body shape, we chose to compare the DNA methylation profile between A and GF-FAT independently in the apples and in the pears. This time, we opted for a threshold of log2-fold change with a *p*-value < 0.001 to discern DMS between the 2 conditions. Only 56 (in pears) and 129 (in apples) CpG sites displayed changes between A-FAT and GF-FAT whereas this increase to 253 (in pears – Supplementary Figure 3A left graph) and 2,912 (in apples – Supplementary Figure 3A right graph) when A and GF criteria were considered in preadipocytes. The lists of DMS in each group are reported in Supplementary Table 3. Almost all of the depot-specific DMS found in pear-shaped subjects (233 from the initial 253) were also detected in apple-shaped subjects (Supplementary Figure 3A). The 2,912 DMS identified in apple women were assigned to 943 unique nearest genes (282 A specific and 661 GF specific). We identified one or more depot specific DMS at proximity of 24 genes that have been previously shown to be differentially expressed between A and G-FAT (Karastergiou et al., 2013; Pinnick et al., 2014; Passaro et al., 2017) (Supplementary Figure 3B). The highest number of DMS between A and GF-preadipocytes were identified nearest genes involved in developmental processes of regionalization and positional identity (HOX genes, transcription factors TBX5, TBX15, PITX1, PITX2, SHOX2; Supplementary Figure 3B). Of note, the TBX15 gene, known to be upregulated in GF-FAT, reported the highest number of hyper methylated sites in A depot compared to GF depot consistent with DMS associated gene repression. This gene was identified as one of the most differentially hypomethylated genes in obese adipose stem cells, and genetic experiments revealed that TBX15 is a regulator of mitochondrial mass in obese adipocytes (Ejarque et al., 2019). Interestingly, pathways enrichment analysis revealed that the 661 nearest genes to the DMS up in GF depot were associated with “Xenobiotic metabolism” and “Estrogen response” (Supplementary Figure 3C). Biogenesis of the oxidative phosphorylation system occurs during adipogenesis (Ryu et al., 2013) and this could lead to activation of genes involved in xenobiotic metabolism to remove xenobiotics generated as products of oxidative metabolism. Increased TBX15 is also potentially associated with mitochondrial mass in GF depot which may reflect a higher capacity of differentiation in the GF compared to the A preadipocytes. In addition, a mouse study showed that increased expression of Estrogen Receptor in adipose tissue is associated with better adipose pad expansion and less inflammation and fibrosis (Blüher, 2013). Interestingly, differential effects of estrogen on upper vs. lower adipose tissue depot has never been reported and merits further investigations. In contrast, the 282 nearest genes to the DMS up in A depot were associated with “Hedgehog signaling” pathway (Supplementary Figure 3C), which was previously shown to inhibit adipogenic differentiation of rodent cells and adipocyte maturation in human mesenchymal stem cells (Fontaine et al., 2008).

## Discussion

DNA methylation has been associated with gene repression related to numerous cellular processes, such as X chromosome inactivation, embryonic development, genomic imprinting, and transposon inactivation (Jones, 2012). As of today, we know that obesity, weight loss intervention and type 2 diabetes influence adipose tissue DNA methylation levels but the specific role of methylation changes relative to adipose tissue function is still unclear and under investigation. Our study revealed for the first time a distinct scWAT methylome profile between apple- and pear-shaped women, suggesting a role of DNA methylation in fat distribution. As age and BMI are shown to be associated with DNA methylation profiles (Farré et al., 2015), the narrow ranges (Table 1) minimize their potential confounding effects. Gene annotation coupled to pathway analysis revealed that the body specific DMS are close to genes involved in cellular communication, cytoskeleton modifications and inflammatory cells recruitment, all crucial actors of tissue development, homeostasis and pathogenesis. Importantly, inflammatory pathway enrichment in apple-shaped women in whole adipose tissue could result from the preferential accumulation of macrophages in adipose depots of apple-shaped women as we previously revealed by immunohistochemistry staining (Divoux et al., 2020). However, genes coding for inflammatory proteins were identified close to body specific DMS in A preadipocytes as well, suggesting that the preadipocytes also directly contribute to the adipose depot inflammatory signature. Altogether, these results are consistent with DMS being associated with differences in adipose tissue phenotype and growth capacity between apple and pear-shaped women. We also examined whether single nucleotide polymorphisms (SNPs) that have been associated with waist to hip ratio were enriched regions of apple-pear DMS. Among the 53 genomic loci previously correlated to fat distribution by SNPs (Lotta et al., 2017), we detected body shape DMS near to only one of them. A more recent paper highlighted 62 SNPs associated with both higher adiposity and lower cardiometabolic risk (Huang et al., 2021). Out of this list, we found only 2 genes with apple specific DMS (ADCY9 in A-depot and AKAP6 in GF-depot), both genes showing correlation with BMI rather than WHR.

The presence of body shape DMS specific to each scWAT (130 in A-FAT and 168 in GF-FAT), indicates that DNA methylation signatures may be a pre-determined, inherent feature of the cells that confers differential gene expression regulation in scWAT depots. In this context, the identification of body shape specific DMS near to genes involved in lipid metabolism only in GF-FAT may contribute to the differential capacity of fat storage between apple and pear-shaped women in their lower body. The particular microenvironment of the A-FAT and GF-FAT [for example relative to inflammation, Extra Cellular Matrix content and angiogenesis capacity (Vogel et al., 2018; Hilton et al., 2019; Divoux et al., 2020)] could be at the origin of depot specific DMS observed in tissue and in cells.

The DMS identified in the whole tissue were mostly preserved in cultured preadipocytes and interestingly, also found in DNA extracted from whole blood. Additional variation may be due to other cell types present in the whole tissue. These results suggest that differential methylation in scWAT is at least part of a broader multi-tissue based long-term epigenetic program that preexists in the body long before the phenotypic differences that define apple vs. pear-shaped body distribution. This also suggests a strong signature of differential DNA methylation which is associated with the apple and pear body shape pattern that is not restricted to adipose tissue. Consistent with the emerging literature of maternal and paternal programming of metabolic disease risk (Hur et al., 2017; Magnus et al., 2018), this may be influenced by maternal or paternal factors that define heritable chromatin patterns that are established early during development. These results also suggest that the metabolic complications associated with apple vs. pear body shape may be at least partically influenced by mechanisms operating outside of the adipose tissue itself.

The presence of the differential methylation signatures in DNA from whole blood also may be leveraged to predict whether a young girl will likely mature into the apple vs. pear body shape long before the differential fat patterning and altered WHR occurs. In addition, our data revealed that isolated preadipocytes are a relatively good *in vitro* model to study the role of DNA methylation in the heterogeneity of fat distribution, as suggested by other publications (Karastergiou et al., 2013; Karpe and Pinnick, 2015).

Comparing the A vs. GF methylated sites independently in apple- and in pear-shaped women, we identified scWAT depot specific DMS only present in apple-shaped women (Supplementary Figure 2A). Intriguingly, we found 20 times more DMS in preadipocytes vs. the whole adipose tissue in apple-shaped women. It is important to note that all the cells were cultured and passaged the same way. Knowing the key role of DNA methylation changes during adipogenesis (Yang et al., 2016), the increasing number of DMS observed in preadipocytes could suggest different level of commitment through the adipogenic lineage between A and GF cells. The reason that these depot-specific DMS were observed only in one group of subjects could result from the distinct micro-environment inherent to the apple fat depot (Vogel et al., 2018; Hilton et al., 2019; Divoux et al., 2020). In addition, the number of unique DMS is more pronounced in GF-preadipocytes, suggesting two hypotheses: (1) More changes occur in the development of GF-adipocytes or/and (2) the GF preadipocytes are more susceptible to environmental variations. Overall, by comparing the A-FAT and GF-FAT methylome, we highlighted major differences in methylation enriched around genes involved in development, suggesting that progenitors from different mesoderm sub-domains may rise to the origin of scWAT and different combinations of TBX, HOX, and PITX genes may at least partially define A and GF-FAT depots. We also found DMRT3 as a gene hypermethylated in A compared to GF-preadipocytes. This gene encodes a transcription factor that was recently shown to be differentially expressed between A-FAT and GF-FAT at baseline and post exercise training, mainly in adipocytes from obese women of African American descent (Nono Nankam et al., 2020). Unlike the homeobox family, *DMRT* family has been less studied in the context of body fat distribution and DMRT3 might be a novel candidate gene involved in “unhealthy” central fat accumulation. We showed that DNA methylation could influence its transcriptional regulation at the preadipocytes state before differentiation into mature adipocytes.

The low concordance between the DMS and gene expression variation in preadipocytes merits further investigation. It is possible that genes associated with DMS are poised to respond to signals associated with excess calorie consumption that drive depot specific gene expression patterns that subsequently influence differential fat accumulation. We reached a similar conclusion in studies of ATAC-seq defined open chromatin regions for GF associated adipocytes (Divoux et al., 2017). It is also possible that DMS influence expression of genes located at remote distances from their location.

## Conclusion

To our knowledge, we revealed for the first-time body shaped specific DNA methylation sites in healthy premenopausal women. Initially identified in scWAT samples, a significant fraction of these sites were also found in whole blood and could serve as a diagnostic predictor of young women who are at increased risk for progressing to the apple body shape with a higher risk of developing obesity related complications. As others previously reported (Pinnick et al., 2014), we highlighted a specific methylation signature between upper and lower scWAT. In our study, these differences were mainly found in apple shaped women. The low concordance observed between the changes in methylation and transcriptional expression may reflect important limitations of our study. Preadipocytes at different passages were used to study the transcriptome and the methylome. Additionally, the DMS were annotated to the closest linearly associated gene, restricting the integration between gene expression and DNA methylation to a simple linear genomic proximity. More accurate integration may require more advanced methods to annotate DMS to specific genes. Current *in vitro* studies of the 3D structure of the chromatin are underway and may help resolve this limitation.

## Data Availability Statement

The datasets presented in this study can be found in online repositories. The name of the repository and accession number can be found below: NCBI GEO database (http://www.ncbi.nlm.nih.gov/geo/) under accession number GSE176603.

## Ethics Statement

The studies involving human participants were reviewed and approved by clinicaltrials.gov, NCT02728635. Registered March 24, 2016, retrospectively registered, https://clinicaltrials.gov/ct2/show/NCT02728635 and clinicaltrials.gov, NCT02226640. Registered 16 May 2014, https://clinicaltrials.gov/ct2/show/NCT02226640. The patients/participants provided their written informed consent to participate in this study.

## Author Contributions

AD designed the experiments, collected human samples, isolated preadipocytes, performed DNA extraction, participated in all data analysis, and wrote the manuscript. AE performed all the DNA methylation analysis. EE performed pathway analysis and figures related to it. KS performed RNA extraction. SS designed and was the principal investigator of the clinical study. TO and SS planned the project. All authors commented on the manuscript, read and approved the final manuscript.

## Conflict of Interest

AE is employed by Prescient Metabiomics Corp. The remaining authors declare that the research was conducted in the absence of any commercial or financial relationships that could be construed as a potential conflict of interest.

## Publisher’s Note

All claims expressed in this article are solely those of the authors and do not necessarily represent those of their affiliated organizations, or those of the publisher, the editors and the reviewers. Any product that may be evaluated in this article, or claim that may be made by its manufacturer, is not guaranteed or endorsed by the publisher.

## Supplementary Material

Supplementary Figure 1 DNA methylation landscape in apple- versus pear-shaped women. CpG sites were mapped to genome regions based on Illumina annotation. We calculated the number of differentially methylated sites (DMS) within the apple (purple bars) and pear (blue bars) groups stratified on functional gene regions. TSS1500; within 1500 basepairs of transcriptional start site (TSS). TSS200; within 200 basepairs of TSS.

Supplementary Figure 2 **(A)** Pathway Analysis of the genes nearest the body shape specific DMS in A cells (A), GF cells (GF) and common in cells from both depots (both). The size of the dots represents gene count. Only pathways with *p*-value < 0.05 are represented. **(B)** Pathway Analysis of the differentially expressed genes between 10 apple- and 7 pear-shaped women in A and in GF depot. The size of the dots represents gene count. Only pathways with *p*-value < 0.05 are represented.

Supplementary Figure 3 Differentially methylated sites between abdominal (A) and gluteofemoral (GF) fat depot in apple and pear-shaped women. **(A)** Volcano plot of differences in DNA methylation between A and GF depot in 7 pear (left side) and 10 apple (right side)-shaped subjects. Each point represents a CpG site significantly differentially methylated with a β-value difference between both depots (methylation difference A *vs.* GF) superior to 0.2. Pink color represents the sites more methylated in A preadipocytes. Blue color represents the sites more methylated in GF preadipocytes. **(B)** The tables report the number of depot specific DMS found at proximity of genes previously reported as differentially regulated between A and GF-FAT. Only the gene with one or more DMS are listed. FC = Fold change of methylation level between A and GF-cells. **(C)** Pathway Analysis of the genes nearest the depot specific DMS in Apple-shaped women, the DMS hypermethylated in A (up_in_A) and hypermethylated in GF (up_in_GF) are represented independently. The size of the dots represents gene count. Only pathways with *p*-value < 0.05 are represented.

Supplementary Table 1 List of differentially methylated sites between 10 Apple and 7 Pear-shaped women in A-FAT, GF-FAT, A-preadipocytes and GF-preadipocytes. Anova, *p*-value < 0.05, (Apple β-value)-(Pear β-value) ≥ 0.2.

Supplementary Table 2 List of differentially expressed genes between Apple and Pear-shaped subjects in abdominal and gluteofemoral scWAT. Deseq2, *p*-value < 0.05.

Supplementary Table 3 List of differentially methylated sites between A and GF in 10 apple-FAT, 7 pear-FAT, 10 apple preadipocytes and 7 pear preadipocytes. Anova, adj *p*-value < 0.001, (A β-value)-(GF β-value) ≥ 0.2.

## Abbreviations

A-FAT: Abdominal fat
DMS: Differentially Methylated Site
GF-FAT: Gluteo-femoral fat
scWAT: subcutaneous White Adipose Tissue
SVF: Stroma Vascular Fraction
WHR: Waist to Hip Ratio.
