# Supplementary figures and images for: DNA Methylation as a Marker of Body Shape in Premenopausal Women
Source: Front Genet. 2021 Jul 29;12:709342. doi: 10.3389/fgene.2021.709342 (PMC8358448; doi:10.3389/fgene.2021.709342)

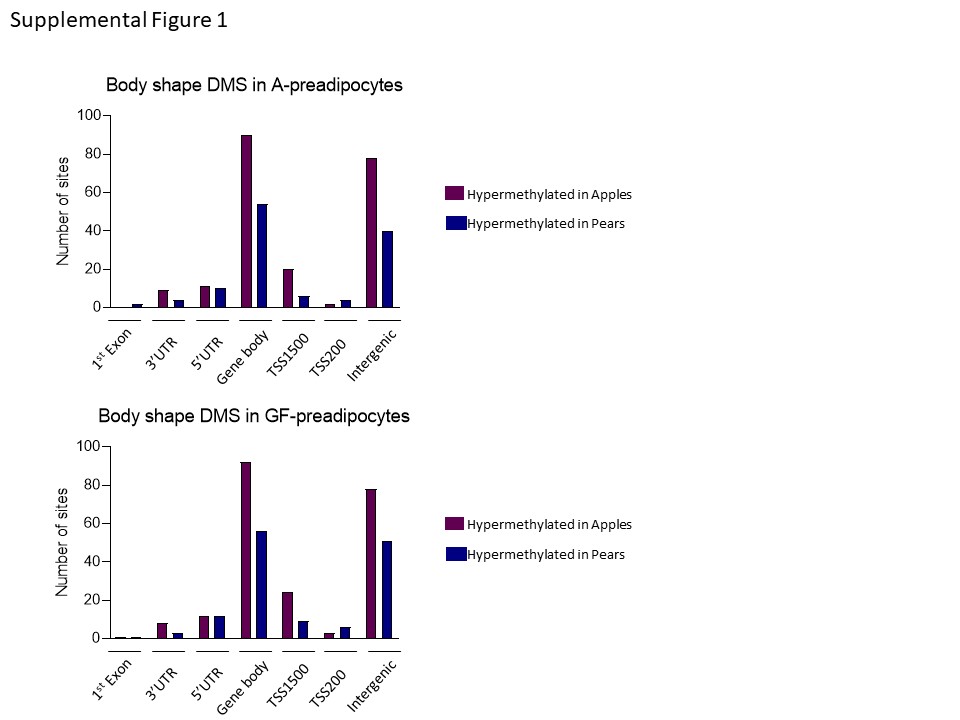

Supplement: Supplementary Figure 1 — DNA methylation landscape in apple- versus pear-shaped women. CpG sites were mapped to genome regions based on Illumina annotation. We calculated the number of differentially methylated sites (DMS) within the apple (purple bars) and pear (blue bars) groups stratified on functional gene regions. TSS1500; within 1500 basepairs of transcriptional start site (TSS). TSS200; within 200 basepairs of TSS. [file Image_1.JPEG]

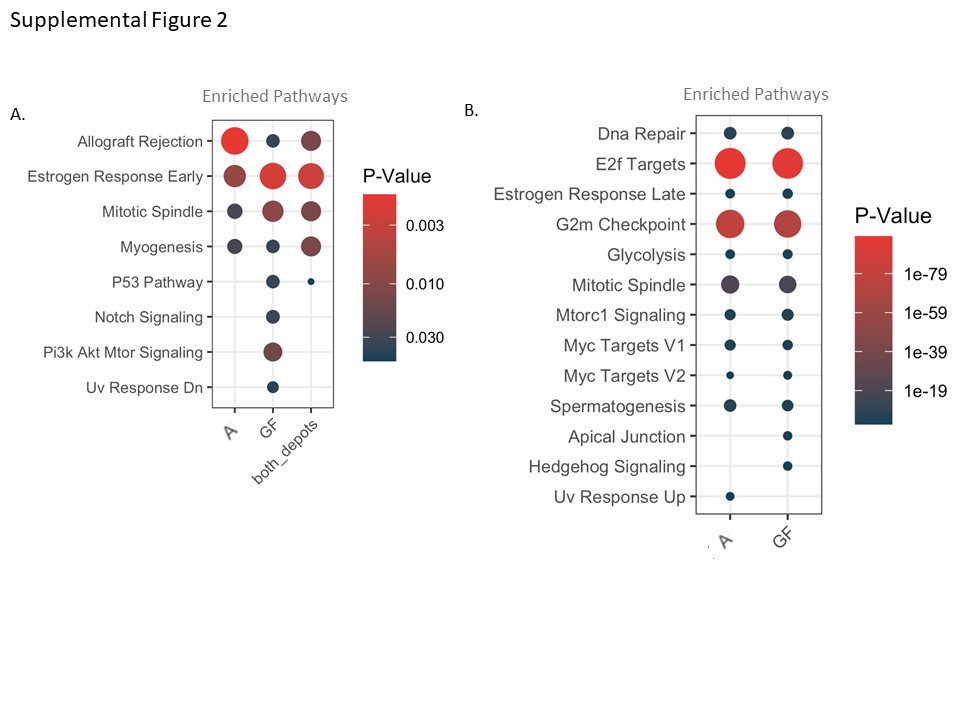

Supplement: Supplementary Figure 2 — (A) Pathway Analysis of the genes nearest the body shape specific DMS in A cells (A), GF cells (GF) and common in cells from both depots (both). The size of the dots represents gene count. Only pathways with p-value < 0.05 are represented. (B) Pathway Analysis of the differentially expressed genes between 10 apple- and 7 pear-shaped women in A and in GF depot. The size of the dots represents gene count. Only pathways with p-value < 0.05 are represented. [file Image_2.JPEG]

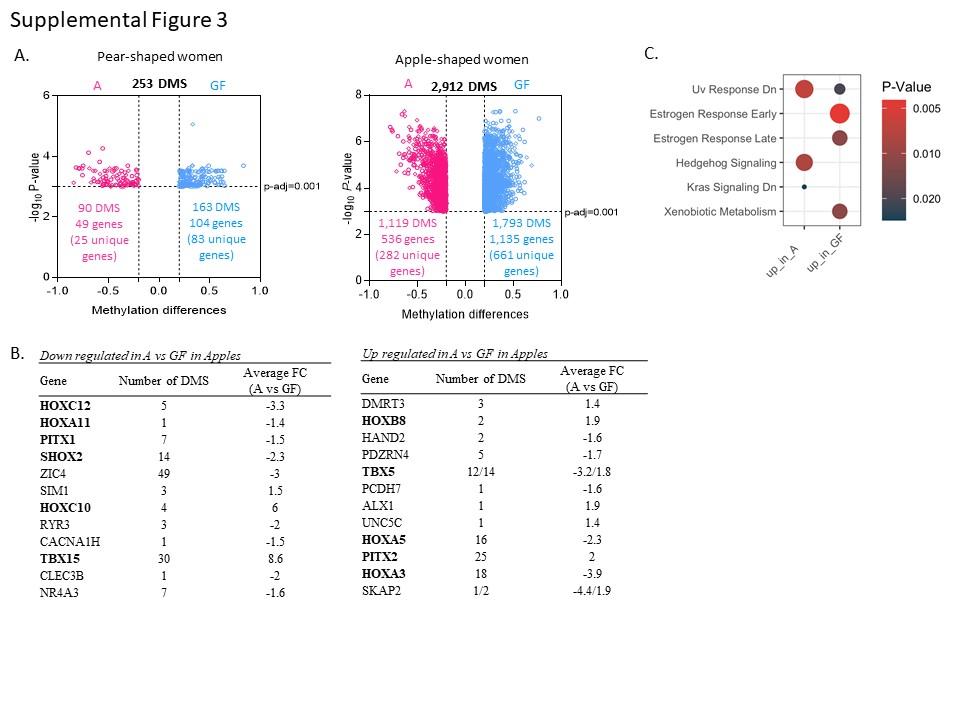

Supplement: Supplementary Figure 3 — Differentially methylated sites between abdominal (A) and gluteofemoral (GF) fat depot in apple and pear-shaped women. (A) Volcano plot of differences in DNA methylation between A and GF depot in 7 pear (left side) and 10 apple (right side)-shaped subjects. Each point represents a CpG site significantly differentially methylated with a β-value difference between both depots (methylation difference A vs. GF) superior to 0.2. Pink color represents the sites more methylated in A preadipocytes. Blue color represents the sites more methylated in GF preadipocytes. (B) The tables report the number of depot specific DMS found at proximity of genes previously reported as differentially regulated between A and GF-FAT. Only the gene with one or more DMS are listed. FC = Fold change of methylation level between A and GF-cells. (C) Pathway Analysis of the genes nearest the depot specific DMS in Apple-shaped women, the DMS hypermethylated in A (up_in_A) and hypermethylated in GF (up_in_GF) are represented independently. The size of the dots represents gene count. Only pathways with p-value < 0.05 are represented. [file Image_3.JPEG]
